# Pleuropulmonary blastoma: a rare clinical image

**DOI:** 10.11604/pamj.2024.48.3.43295

**Published:** 2024-05-02

**Authors:** Ashwin Karnan

**Affiliations:** 1 Department of Respiratory Medicine, Jawaharlal Nehru Medical College, Datta Meghe Institute of Higher Education and Research, Sawangi (Meghe), Wardha, Maharashtra, India

**Keywords:** Cough, cyst, pleura, effusion, rhabdomyosarcoma

## Image in medicine

A 13-year-old boy presented to the outpatient department with complaints of breathing difficulty for the past 2 months. Magnetic resonance imaging of the thorax showed a solid cystic mass lesion in the right hemithorax of size 8.9 x 10.7 cm with mild pleural effusion with mass effect shifting the major vessels to the left side. Computed tomography-guided biopsy was done which showed variable thickened nodule-like areas with both single cells and cohesive aggregates with positive stains for vimentin and cytokeratin. A diagnosis of pleuropulmonary blastoma was made. The patient underwent surgical resection and is currently on follow-up. Pleuropulmonary blastomas are rare and aggressive childhood intrathoracic tumors common in children less than 6 years of age. It may be of three types- type I (cystic), type II (mixed), or type III (solid). Clinical presentation includes shortness of breath, chest pain, cough, and hemoptysis. Tumor size >5 cm with pleural or mediastinal invasion has a poor prognosis. Surgical resection, postoperative radiotherapy, and chemotherapy are available.

**Figure 1:**
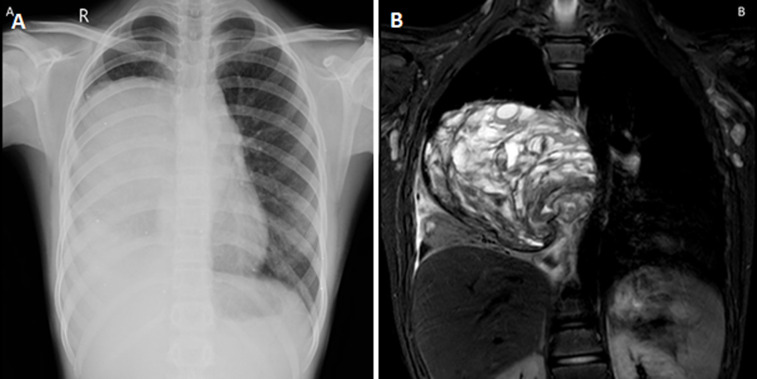
A) chest X-ray of the patient showing homogenous opacity in the right lung; B) MRI of the thorax showing solid cystic lesion in the right hemithorax with mild pleural effusion

